# Agro-Alimentary Potential of the Neglected and Underutilized Local Endemic Plants of Crete (Greece), Rif-Mediterranean Coast of Morocco and Tunisia: Perspectives and Challenges

**DOI:** 10.3390/plants10091770

**Published:** 2021-08-25

**Authors:** Mohamed Libiad, Abdelmajid Khabbach, Mohamed El Haissoufi, Ioannis Anestis, Fatima Lamchouri, Soumaya Bourgou, Wided Megdiche-Ksouri, Zeineb Ghrabi-Gammar, Vasileios Greveniotis, Ioannis Tsiripidis, Eleftherios Dariotis, Maria A. Tsiafouli, Nikos Krigas

**Affiliations:** 1 Laboratory of Ecology, Systematics and Biodiversity Conservation (LESCOBIO), Department of Biology, Faculty of Sciences, Abdelmalek Essaâdi University, B.P. 2121, M’Hannech II, Tetouan 93000, Morocco; 2 Laboratory of Natural Substances, Pharmacology, Environment, Modelling, Health and Quality of Life (SNAMOPEQ), Polydisciplinary Faculty of Taza, Sidi Mohamed Ben Abdellah University, B.P. 1223, Taza Gare, Taza 35000, Morocco; khamajid@hotmail.com (A.K.); mohamed.elhaissoufi1@usmba.ac.ma (M.E.H.); fatima.lamchouri@usmba.ac.ma (F.L.); 3 Laboratory of Biotechnology, Conservation and Development of Natural Resources (BCVRN), Department of Biology, Faculty of Sciences Dhar El Mahraz, Sidi Mohamed Ben Abdellah University, B.P. 1796, Fès-Atlas 30003, Morocco; 4 Department of Ecology, School of Biology, Aristotle University of Thessaloniki, 54124 Thessaloniki, Greece; ganestis3@gmail.com (I.A.); tsiafoul@bio.auth.gr (M.A.T.); 5 Laboratoire des Plantes Aromatiques et Médicinales, Centre de Biotechnologie de Borj-Cédria, B.P. 901, Tunis 2050, Tunisia; bourgousoumaya@yahoo.com (S.B.); ksouriwided@yahoo.fr (W.M.-K.); 6 Institut National Agronomique de Tunisie, Université de Carthage, 43 Avenue Charles Nicolle, Cité Mahrajène, Tunis 1082, Tunisia; zghrabi@yahoo.fr; 7 Laboratoire de Recherche Biogéographie, Climatologie Appliquée et Dynamiques Environnementales (BiCADE 18ES13), Faculté des Lettres des Arts et des Humanités de Manouba, Université de la Manouba, Campus Universitaire de la Manouba, Manouba 2010, Tunisia; 8 Institute of Industrial and Forage Crops, Hellenic Agricultural Organization Demeter, 41335 Larisa, Greece; vgreveni@mail.com; 9 Department of Botany, School of Biology, Aristotle University of Thessaloniki, 54124 Thessaloniki, Greece; tsiripid@bio.auth.gr; 10 Institute of Plant Breeding and Genetic Resources, Hellenic Agricultural Organization Demeter, Thermi, P.O. Box 60458, 57001 Thessaloniki, Greece; eleftheriosdariotis@yahoo.com

**Keywords:** climate change, food security, Mediterranean countries, sustainable exploitation, phytogenetic resources

## Abstract

The neglected and underutilized plants (NUPs) could become alternative food sources in the agro-alimentary sector, enriching human and animal diets, offering the opportunity for sustainable exploitation, resilience to climate change, and production with resistance to pests and diseases. In the Mediterranean countries, these valuable resources are threatened by climate change, overexploitation, and/or monoculture. In this framework, we evaluated 399 local endemic NUPs of Crete (Greece), the Mediterranean coast, Rif of Morocco, and Tunisia, regarding their agro-alimentary potential, and assessed their feasibility and readiness timescale for sustainable exploitation with own previously published methodology. The methodological scheme was developed by experts in co-creative workshops, using point-scoring of seven attributes to evaluate the potential of the targeted NUPs in the agro-alimentary. Our results showed a diversity of promising local endemic NUPs of different families in the studied regions (Lamiaceae members are prominent), and we outlined the cases of 13 taxa with the highest optimum scores of agro-alimentary potential (>70%). Despite the diversity or the promising potential and current ex-situ conservation efforts to bridge gaps, our study indicated that only a few cases of Cretan local endemic NUPs can be sustainably exploited in the short-term. However, it is argued that many more local endemic NUPs can easily follow sustainable exploitation schemes if specific research gaps are bridged. Since NUPs can help to increased diversification of food production systems by adding new nutritional/beneficial species to human and animal diets, basic and applied research, as well as market and stakeholder attraction, is suggested as prerequisite to unlock the full potential of the focal endemic NUPs in the agro-alimentary sector.

## 1. Introduction

Plants play a great role in human life. It is estimated that at least 31,128 plant species are currently used worldwide, and 17.79% are utilized for human nutrition [1]. Despite this diversity, only 30 plant species (including major staple crops) provide 95% of dietary energy or protein to feed the world [2,3]. However, regional studies in the Mediterranean countries highlight that more than 52% of the wild harvested plants can be used for agro-alimentary purposes [4,5]. Despite the high scale of use of plants in local Mediterranean economies, food resources are also affected by climate change, monocultures, and/or overexploitation [6]. To mitigate the effect of climate change and the degradation of land and water resources, it becomes urgent to engage improved crops and new species that are adapted to difficult environments and can increase the overall productivity and stability of agro-ecosystems [7]. Hence, there is need to change current agricultural practices and promote novel crops that are more resilient to climate change challenges, such as the neglected and underutilized plant species (NUPs). NUPs have an important role to play in diversifying and advancing agricultural development beyond the Green Revolution model by improving the yields of staple crops and by introducing new valuable crops to enrich human and animal nutrition [8]. These NUPs present tremendous opportunities for fighting poverty, hunger, malnutrition, and, at the same time, can increase the development of subsistence economies in local/regional scales [8,9,10,11]. 

The implication of citizens (or consumers), scientists, entrepreneurs, producers, market specialists and stakeholders, and the establishment of targeted or applied research and development programs can act as key drivers to promote sustainable food systems, especially regarding NUPs [8,12,13,14]. The instating of NUPs as alternative food sources in the agro-alimentary sector would depend on the availability of information describing their agronomical aspects, water-use, and possible drought tolerance [15]. There are some NUPs that could contribute to the food security of rural and urban people in India and some countries of South America, e.g., some Andean grains such as quinoa (*Chenopodium quinoa* Willd.) and cañahua (Bolivian name, known as cañihua in Peru) *Chenopodium pallidicaule* Aellen [8], and minor millets such as foxtail millet *Setaria italica* (L.) P. Beauv., proso millet *Panicum miliaceum* L., finger millet *Eleusine coracana* (L.) Gaertn, kodo millet *Paspalum scrobiculatum* L., little millet *Panicum sumatrense* Roth, and barnyard millet *Echinochloa colonum* (L.) Link [11]. In the Mediterranean Basin, the use of resistant NUPs to pests, diseases and extreme environmental factors (e.g., *Cistus ladanifer* L.) may be a viable solution for cultivations in poor and degraded soils. In addition, this species reveals interesting aptitudes that can be applied to food, pharmaceutical, phytochemical, and biofuel industries [16]. 

In both developed and developing countries, the increased demand from consumers for diversity and novelty in modern foods is currently creating new market niches for NUPs [7] due to their high content in vitamins, micronutrients, proteins, and other beneficial compounds [7,9,10]. Consequently, the introduction of NUPs in agro-alimentary production systems could play a strategic role in improving many dimensions of livelihoods and well-being, and therefore may represent an important source of household income, also encouraging women empowerment [7,9,10,11,17]. Conversely, better marketing and consumer awareness of the benefits associated with NUPs could play a critical role in their sustainable exploitation [12]. 

In an increasingly human-dominated world, conservation of species in the wild is (or should be) a top-priority [18], and the precedence should be given to the local endemic plants of biodiversity-rich regions [19,20]. Typically, the criteria adopted for conservation prioritization involve aspects of the geographic distribution, endemism, and the threats affecting each evaluated taxon [19,20], including harvesting from the wild. However, to date, some thousands of medicinal-aromatic plants, as well as the vast majority of the wild edible greens, are still collected directly from the wild [21], depleting their natural potential. In Mediterranean areas, the possibility of first genotypic selection, targeted and/or improved pilot cultivation, and new processing techniques for specific applications gives the NUPs the potential to be used as a valuable novel resource [16]. Furthermore, high education status and primary occupation of the household head may also have a major role for the conservation of NUPs at local scales [14].

Although NUPs are not intended to replace staple crops, crop diversification is envisaged as one of the best means to ensure sustainable agricultural production systems [3,17], by growing NUPs as part of crop rotation systems or by inter-cropping them with other crops. This practice could protect and enhance agro-biodiversity at field level and may disrupt the cycle of some pests and diseases [3]. The sustainable promotion of NUPs requires better understanding as well as improved marketing and high consumer awareness regarding the benefits associated with selected NUPs [12], while scientific research including agronomical aspects, breeding potential, post-harvest handling, and value chain establishment may bridge existing gaps, linking valuable local resources with farmers and markets [15,22]. This is the context of the current study. Our investigation aims at exploring and documenting for the first time the potential of the single-country/single-region endemic plants of Crete (Greece), Mediterranean Coast and Rif of Morocco, and Tunisia in the agro-alimentary sector, with the scope to identify the actions needed to remove barriers and bridge gaps regarding their sustainable exploitation. 

## 2. Results

### 2.1. Cluster Analysis of Agro-Alimentary Attributes and of Focal Taxa

**Figure 1:**
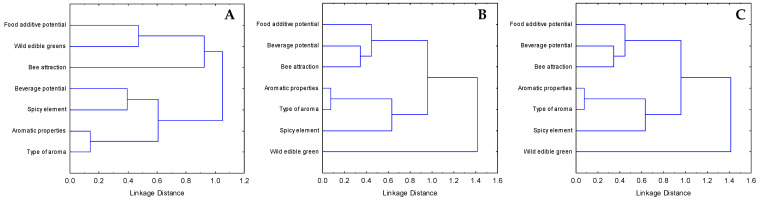
Graph of hierarchical clustering of the agro-alimentary attributes (complete linkage, 1-Pearson r distance) based on the score values of the local endemic plants of (**A**) Crete, (**B**) Mediterranean coast and Rif of Morocco, and (**C**) Tunisia.

The results of the hierarchical cluster analyses of agro-alimentary attributes (Figure 1) showed that, in the case of Crete, the attribute ‘food additive potential’ was grouped together with the attribute ‘wild edible greens’ next to the ‘bee attraction’ attribute. The ‘beverage potential’ and ‘spicy element’ as attributes formed another subgroup, while these were clustered together with the subgroup ‘aromatic properties’ and ‘type of aroma’.

In the case of Tunisia, as well as the Mediterranean coast and Rif of Morocco, a similar pattern was observed; the attributes ‘beverage potential’ and ‘bee attraction’ formed a subcluster, and they were clustered together with the attribute ‘food additive potential’. The attributes ‘type of aroma’ and ‘aromatic properties’ were closely linked together and formed a cluster with the attribute ‘spicy element’, as in the case of Crete. Finally, the attribute ‘wild edible greens’ was not linked to any other attribute. 

### 2.2. Diversity of Local Endemic NUPs

The evaluation of the potential of the focal endemic NUPs in the agro-alimentary sector showed that Lamiaceae family members (26 taxa) are mostly represented in the top 15 taxa of each of the three studied regions, followed by members of Asteraceae (5 taxa), Liliaceae (4 taxa in the case of Crete), Caryophyllaceae, Gentianaceae, Plumbaginaceae, and Rubiaceae (2 taxa each), as well as Apiaceae and Pinaceae (1 taxon each). Regarding the top 15 evaluated NUPs of each region, Lamiaceae members prevail again in the case of the Mediterranean coast and Rif of Morocco (11 taxa), followed by Crete (9 taxa) and Tunisia (6 taxa). 

### 2.3. Agro-Alimentary Potential of the Focal Local Endemic NUPs

Supplementary Material S1 provides examples of scoring of taxa per attribute and the evaluation of the agro-alimentary potential (Level I) of the focal local endemic taxa of the studied regions is presented in Supplementary Material S2 as percentages of the maximum possible scores achieved.

#### 2.3.1. Local Endemic Plants of Crete

**Figure 2:**
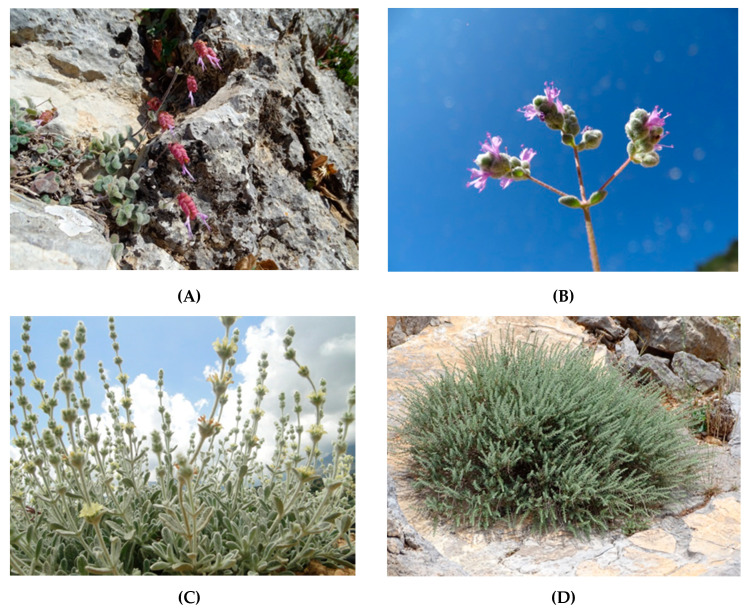
Four top-evaluated Cretan endemic plants of Lamiaceae family, in terms of maximum possible score achieved regarding their agro-alimentary potential (Photos: N. Krigas). (**A**): *Origanum dictamnus*, (**B**): *Origanum microphyllum*, (**C**): *Sideritis syriaca* subsp. *syriaca*, (**D**): *Thymbra calostachya*. For these plants, propagation material has been collected from the wild to allow ex-situ conservation and propagation-cultivation trials at the Institute of Plant Breeding and Genetic Resources, Hellenic Agricultural Organization Demeter.

**Figure 3:**
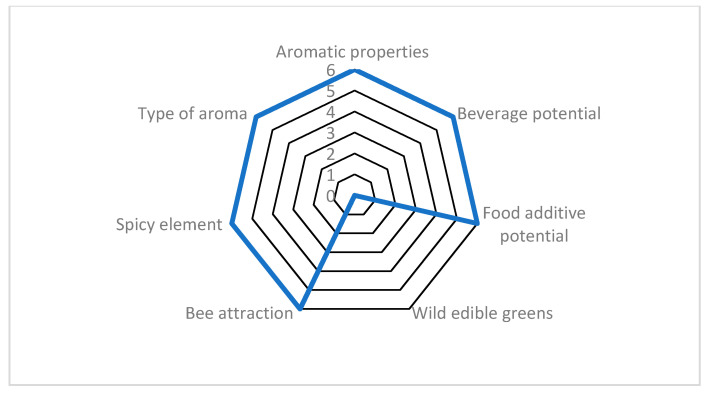
Evaluation example of *Origanum dictamnus* (Cretan endemic) scored for seven agro-alimentary attributes, reaching 85.7% of the optimum possible score. This example is hierarchically ranked in the highest (>70%) class. For attributes and scoring, see Section 4.

Among the Cretan local endemics, the four highest evaluated taxa (Figure 2) were *Origanum dictamnus* L. (85.71%), *Origanum microphyllum* (Benth.) Vogel, *Sideritis syriaca* L. subsp. *syriaca* (80.95 % each), and *Thymbra calostachya* (Rech. f.) Rech. f. (71.42%); these plants showed a very interesting agro-alimentary potential. The scoring of *Origanum dictamnus* (85.71%) is illustrated in Figure 3. In total, three taxa [hierarchically: *Helichrysum doerfleri* Rech. f., *H. heldreichii* Boiss., and *Calamintha cretica* (L.) Lam.] ranked in above-average to high positions with scores 55–70%. Overall, nine taxa ranked above-average with scores 50–55%, i.e., the wild garlic or wild onion plants *Allium bourgeaui* Rech. f. subsp. *creticum* Bothmer, *A. circinnatum* Sieber subsp. *circinnatum*, *A. dilatatum* Zahar., and *A. platakisii* Tzanoud. and Kypr., *Micromeria hispida* Boiss. and Heldr. ex Benth. and *Micromeria sphaciotica* Boiss. and Heldr. ex Benth. (54.76% each). Moreover, another 20 taxa ranked in below-average to low positions with scores 35–50%. For 157 taxa, the scores ranked comparatively very low (<35%), and the lowest value was assigned to 30 taxa due to zero values, e.g., *Carex cretica* Gradst. and J. Kern (Supplementary Material S2).

#### 2.3.2. Local Endemic Plants of the Mediterranean Coast-Rif of Morocco

**Figure 4:**
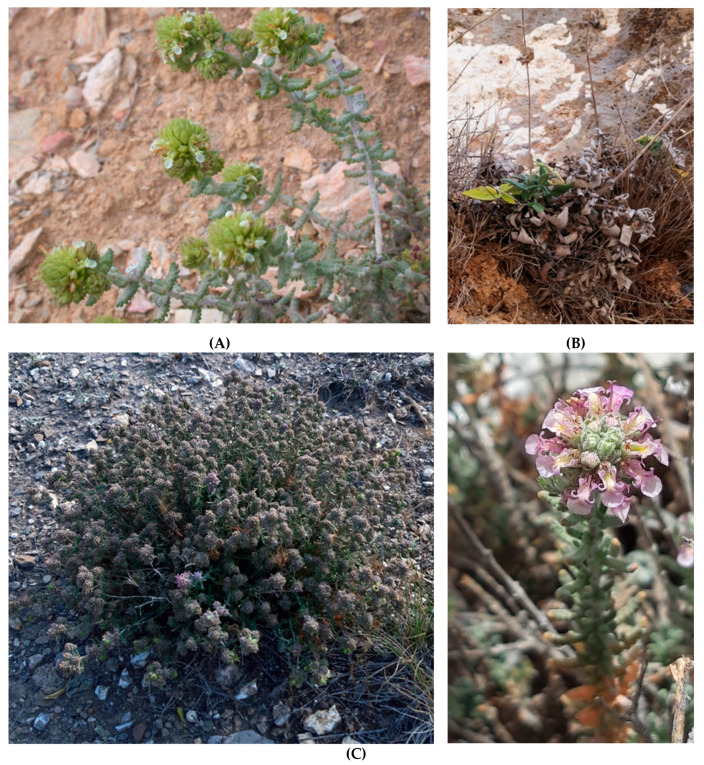
Top-evaluated endemic plants of Mediterranean coast and Rif of Morocco in terms of maximum possible score achieved regarding their agro-alimentary potential. (**A**): *Teucrium huotii* (Photo: M. Rouviere, http://www.ville-ge.ch/musinfo/bd/cjb/africa/details.php?langue=fr&id=145076; accessed on 24 August 2021), (**B**): *Salvia interrupta* subsp. *paui* (Photo: A. Homrani Bakali, https://www.teline.fr/; accessed on 24 August 2021), (**C**): *Teucrium gypsophilum* (Left photo: F. Lamchouri; right photo: A. Khabbach). Propagation material has been collected from the wild for *Teucrium gypsophilum* to allow ex-situ conservation and propagation-cultivation trials at the Institute of Plant Breeding and Genetic Resources, Hellenic Agricultural Organization Demeter.

**Figure 5:**
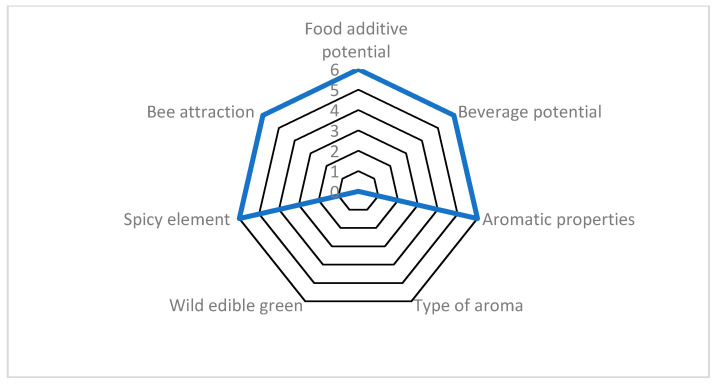
Evaluation example of *Centaurium eythraea* subsp. *bifrons* (endemic to the Mediterranean coast and Rif of Morocco) scored for seven agro-alimentary attributes, reaching 71.4% of the optimum possible score. This example is hierarchically ranked in the highest (>70%) class. For attributes and scoring, see Materials and methods.

The six highest-evaluated North Moroccan taxa were *Centaurium erythraea* Rafn subsp. *bifrons* (Pau) Greuter, *Teucrium afrum* (Emb. and Maire) Pau subsp. *rubriflorum* (Pau and Font Quer) Castrov. and Bayon, *T. grosii* Pau, *T. gypsophilum* Emb. and Maire, *T. huotii* Emb. and Maire and *T. rotundifolium* Schreb. subsp. *sanguisorbifolium* (Pau and Font Quer) E.Cohen (each 71.43%), showing a very interesting agro-alimentary potential (Figure 4). The scoring of *Centaurium erythraea* subsp. *bifrons* (71.43%) is illustrated in Figure 5. In total, four taxa, namely *Teucrium chlorostachyum* Pau and Font Quer subsp. *chlorostachyum, T. chlorostachyum* subsp. *melillense* (Maire) El Oualidi, Mathez and T. Navarro, *T. rifanum* (Maire and Sennen) T. Navarro and El Oualidi, *Salvia interrupta* Schousb. subsp. *paui* (Maire) Maire, ranked in above-average to high positions, with scores > 55–70%. Overall, eight taxa ranked in lower to average positions with scores 35–50%, i.e., *Abies marocana* Trab. (47.62%), *Centaurium barrelieroides* Pau (45.24%), *Marrubium fontianum* Maire and *M. heterocladum* Emb. and Maire (42.86% each), *Anthemis mauritiana* Maire and Sennen subsp. *mauritiana, Origanum elongatum* (Bonnet) Emb. and Maire, *Stachys fontqueri* Pau and *Vicia cedretorum* Font Quer (38.10% each). For 67 taxa, the scores ranked comparatively very low (<35%), and the lowest positions were assigned to 9 taxa with the score zero values, e.g., *Hemicrambe fruticulosa* Webb (Supplementary Material S2).

#### 2.3.3. Local Endemic Plants of Tunisia

**Figure 6:**
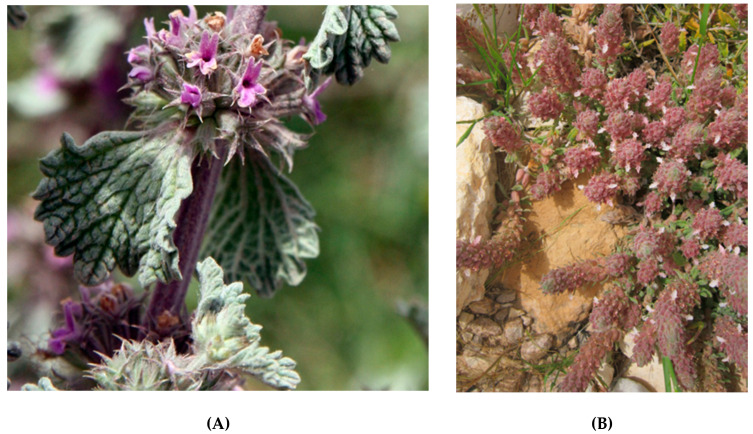

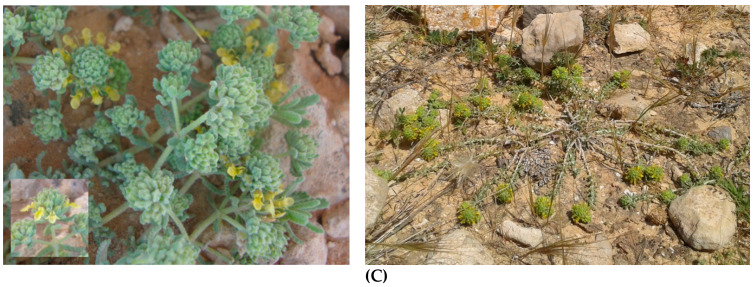
Three top-evaluated Tunisian endemic plants of Lamiaceae family, in terms of maximum possible score, achieved regarding their agro-alimentary potential. (**A**): *Marrubium aschersonii* (Photo: G. Dakhlia), (**B**): *Teucrium alopecurus* (Photo: Z. Ghrabi-Gammar), (**C**): *Teucrium luteum* subsp. *gabesianum* (Left photos: Z. Ghrabi-Gammar; right photo: S. Bourgou). For these plants, propagation material has been collected from the wild to allow ex-situ conservation and propagation-cultivation trials at the Institute of Plant Breeding and Genetic Resources, Hellenic Agricultural Organization Demeter.

**Figure 7:**
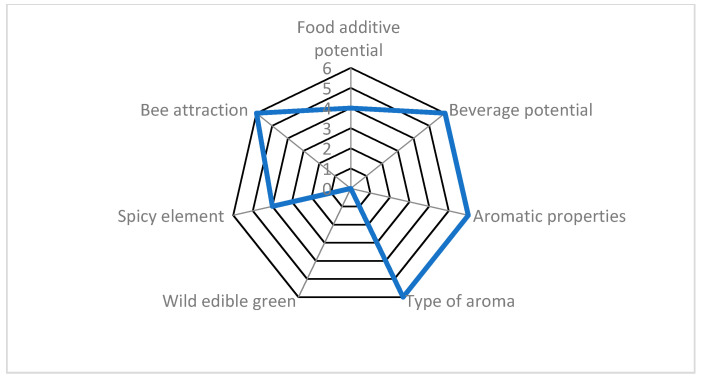
Evaluation example of *Marrubium aschersonii* (local Tunisian endemic) scored for seven agro-alimentary attributes, reaching 76.2% of the optimum possible scores. This example is hierarchically ranked in the highest (>70%) class. For attributes and scoring, see Materials and methods.

Three Tunisian endemic taxa were ranked in the highest positions (>70%) regarding the general potential in the agro-alimentary sector, i.e., *Marrubium aschersonii* Magnus (76.19%), *Teucrium alopecurus* de Noë and *T. luteum* (Mill.) Degen subsp. *gabesianum* (S.Puech) Greuter and Burdet ex Greuter (71.43% each, Figure 6). The scoring of *Marrubium aschersonii* (76.19%) is illustrated in Figure 7. Only *Artemisia campestris* L. subsp. *cinerea* Le Houér was ranked in the above average to high positions (66.67%). Overall, eight taxa ranked in lower to average positions, with scores 40.48–47.62%, e.g., *Teucrium nablii* S. Puech, *T. radicans* Bonnet and Barratte and *T. sauvagei* Le Houér. (47.62% each), *Calendula suffruticosa* Vahl subsp. *suffruticosa, Dianthus cintranus* Boiss. and Reut. subsp. *byzacenus* (Burollet) Greuter and Burdet, *D. rupicola* Biv. subsp. *hermaeensis* (Coss.) O. Bolòs and Vigo (42.86% each), *Galium afropusillum* Ehrend., and *G. olivetorum* Le Houér. (40.80% each). For another 70 taxa, the scores ranked comparatively very low (<35%). The lowest score was assigned to 11 taxa (2.38% each, see Supplementary Material S2).

## 3. Discussion

### 3.1. Agro-Alimentary Potential of the Studied Local Endemic NUPs

Previous studies report that the unsustainable collection and trade directly from wild plant populations, when coupled with absence of knowledge on ex-situ conservation of propagation materials, may considerably decrease the availability of local phytogenetic resources, and this detrimental effect can lead in turn to increased prices of plant products [23,24]. Unfortunately, many plant materials, especially aromatic-medicinal plants and wild edible greens are still gathered in an unsustainable way; they are purchased directly from the wild as raw materials and are channelled for industrial use [21].

In the frame of the sustainable use of plant genetic resources as an essential component for food security and food diversity in the face of climate change, the Neglected and Underutilized Plant species (NUPs) are considered as promising alternative crops, if domesticated and sustainably used by marginalized farmers in local economies [8,11,22]. Previous studies estimate that the domestication and promotion of NUPs, such as the wild fennel in Morocco and amaranth in Ecuador, could increase the household annual income by 75% and 20%, respectively [8]. In addition, the NUPs could generate up to 62% of a farmer’s annual income (1125 US$), e.g., *Gnetum* spp. in Nigeria and Cameroun [23]. Furthermore, the promotion of NUPs could contribute to their conservation and the maintenance of the associated indigenous knowledge, through wider use of their diversity, adoption of best cultivation practices, development of improved varieties, dissemination of high-quality seed, and capacity development [25].

To date, only some degree of attention has been given to NUPs, prioritizing members of Amaranthaceae or Poaceae [11,25]. However, the focus on local endemic NUPs of different regions is still very limited [2,3,22]. Local endemic NUPs are unfortunately plant species associated with limited and fragmented knowledge, usually attracting the attention of researchers or hobbyists but rarely that of citizens, politicians, and stakeholders [26,27]. The associated knowledge gaps and the research needs for most of these endemic NUPs are immense [9]. However, at least 43 local endemic NUPs of Crete (Greece), Rif and Mediterranean coast of Morocco, and Tunisia are currently traded in high prices worldwide, mainly due to their ornamental-horticultural value [22,28,29,30], and many of them have a very interesting potential in specific subsectors of the ornamental-horticultural industry [22]. The high market value and extant international trade of these NUPs suggest that at least some dozens of the focal NUPs studied herein are well-known, appreciated, and used mainly for ornamental-horticultural purposes. Hence, these commercial channels in place can be exploited to some extent to introduce adequately some of these NUPs (or other NUPs) in the agro-alimentary market. This could certainly be the case when such local endemic NUPs of ornamental-horticultural value also have a very interesting agro-alimentary potential. For example, the local Cretan endemic *Petromarula pinnata* (L.) A. DC. (Campanulaceae), is traditionally consumed in Crete as a wild edible green locally called ‘petrofilia’ (literally meaning rock-dwelling) or ‘petromaroulida’ (meaning rock-lettuce, thus alluring to its value as wild-growing fresh salad plant). 

This study evaluated, for the first time, the agro-alimentary potential of the local endemic NUPs of Greece, Northern Morocco (Mediterranean Coast and Rif), and Tunisia and provided ranking of their potential (top-evaluated cases), thus allowing identification of the most interesting/promising cases of local endemic NUPs per country/region. Our study showed that the Lamiaceae local endemic NUPs (26 taxa) are most represented in the top 15 cases of each of the three studied regions, and thus should be considered quite promising in the agro-alimentary sector. Additionally, local endemic NUPs of Asteraceae (5 taxa), Liliaceae (4 *Allium* taxa in the case of Crete), or NUP members of another six families (Caryophyllaceae, Gentianaceae, Plumbaginaceae, Rubiaceae, Apiaceae, and Pinaceae) are also promising in the agro-alimentary sector. Due to their richness in volatile constituents and/or nutritional elements, these local endemic NUPs represent cases of taxa that could be used potentially as food additives and/or wild edible greens and/or for food flavouring as spicy elements such as members of the genera *Lactuca, Malva, Muscari*, *Rumex, Silene, Sanguisorba, Tragopogom, Thymbra* etc. (Supplementary material S1 and references therein), or are indeed used traditionally in local scales as food additives and/or wild edible greens and/or for food flavouring as spicy elements, e.g., the local endemic NUPs belonging to the genera *Allium*, *Campanula, Centaurea, Cotoneaster, Crepis, Hypochaeris, Origanum, Onopordum, Petromarula, Sonchus,* etc. (Supplementary material S1 and references therein) and/or for beverage preparations (e.g., *Sideritis* spp., *Origanum* spp., *Salvia* spp., *Teucrium* spp., *Thymbra* spp.). In the same fashion, many studies underline, nowadays, that the NUPs may have very high nutrient content or nutraceutical values, and consequently, they are often considered as ‘superfoods’ [7,12]. Recently, this has been the case of *Origanum dictamnus* (local Cretan endemic, with approved medicinal properties by the European Medicines Agency, www.ema.eu; accessed on 24 August 2021), for which a functional food potential has been thoroughly documented [31]. 

Apart from the above-mentioned, which refer to human nutritional/beneficial values, many of the evaluated local endemic NUPs can naturally attract pollinators and may feed bee populations (Supplementary Material S1), such as the NUP members of the families Lamiaceae, Dipsacaceae, and Asteraceae. Furthermore, many local endemic NUPs of the studied regions (Supplementary Material S2) belong to the top 10 families of the Mediterranean region with highly palatable plant species for grazing/foraging livestock [32,33], i.e., Asteraceae (30 Cretan, 15 north Moroccan, and 4 Tunisian local endemic NUPs), Poaceae (5 Cretan, 5 north Moroccan, and 4 Tunisian local endemic NUPs), Fabaceae (12 Cretan, 14 north Moroccan, and 5 Tunisian local endemic NUPs), Amaranthaceae (-), Brassicaceae (12 Cretan, 5 north Moroccan, and 2 Tunisian local endemic NUPs), Boraginaceae (1 north Moroccan and 6 Cretan local endemic NUPs), Caryophyllaceae (30 Cretan, 8 north Moroccan, and 3 local endemic Tunisian NUPS), Lamiaceae (14 Cretan, 16 north Moroccan, and 8 Tunisian local endemic NUPs), Apiaceae (7 Cretan, 4 north Moroccan, and 2 Tunisian local endemic NUPs), and Cistaceae (1 north Moroccan and 2 Tunisian local endemic NUPs). Since the local endemic NUPs of these families (n = 215 taxa; 53.88% of the focal NUPs) are naturally co-occurring in Cretan, Tunisian, or north Moroccan landscapes, together with other commonplace highly palatable members of the same families with more abundant populations, it is quite probable that these local endemic NUPs are also naturally preferred by foraging livestock at local scales due to their similar nutritional value for livestock feeding. This aspect brings into light another important agro-alimentary aspect related to the neglected foraging value of the local endemic NUPs. Certainly, the palatability of the local endemic NUPs of the studied regions could be further studied and documented appropriately, and selected local endemic NUPs could prove to be worth of propagation and cultivation at large-scales for forage/fodder of stabling livestock.

The diversity of unique Mediterranean NUPs with interesting agro-alimentary potential, as evaluated in this study, was further assessed in terms of estimated feasibility and readiness timescale for sustainable exploitation.

### 3.2. Sustainable Exploitation Feasibility of the Focal NUPs in the Agro-Alimentary Sector

Our previous study showed that there is only compromised feasibility in terms of sustainable exploitation [22] regarding the top 15 Moroccan (Mediterranean coast and Rif) endemics evaluated herein for their agro-alimentary potential, i.e., no taxon is evaluated in the highest class (>70%) or in above-average to high positions (>55–70%). *Abies marocana* (43.06%) and *Centaurium erythraea* subsp. *bifrons* (40.28%) are ranked below-average in terms of sustainable exploitation feasibility. Overall, the majority (13) of the top 15 taxa in agro-alimentary potential are ranked in lower positions in terms of exploitation feasibility (<35%). The same applies for the top 15 Tunisian endemics with interesting agro-alimentary potential; none of the Tunisian local endemic NUPs were evaluated as feasible in terms of sustainable exploitation (>70%) and no taxon is ranked in above-average to high positions (>55–70%), or even in average positions (>50–55%). Only *Artemisia campestris* subsp. *cinerea* is ranked marginally average (50%). The majority (13) of the top-evaluated taxa with agro-alimentary interest ranked in low positions (<35%) in terms of sustainable exploitation feasibility. The above findings mainly reflect the extant considerable research gaps such as absence of propagation and cultivation techniques in place, unavailability of propagation material, and compromised stakeholder interest, which actually hinder any kind of exploitation [22], see also Supplementary material S2. To justify this trend, a previous study [34] highlights, in a general way, the absence of horticultural experience regarding the local endemic NUPs of North Morocco and Tunisia, documenting that only a very small number of these local endemic plants are currently found under *ex-situ* conservation in botanic gardens and seed banks worldwide. This trend is in contrast with the comparatively higher number of local endemic NUPs of Crete that are currently in electronic trade worldwide [29].

Among the top 15 Cretan endemics taxa of the agro-alimentary sector, *Origanum dictamnus* is the most promising case, also achieving the highest score in terms of sustainable exploitation feasibility (91.67%). Thus, there are extant value chains and sustainable commercial exploitation at least in Crete where it is endemic and is locally cultivated [28,35,36], almost just as with any other crop [22]. In the same line, another five Cretan endemic taxa that ranked in above-average to high positions in terms of sustainable exploitation feasibility with scores >55.6–69.4% (hierarchically: *Calamintha cretica, Sideritis syriaca* subsp. *syriaca, Nepeta sphaciotica* P. H. Davis, *Helichrysum heldreichii, Thymbra calostachya*), as well as *Origanum microphyllum* that ranked in the above average class (50–55%) with score 52.78%, can also have the chance to become medicinal-aromatic crops in the future. Among them, *Sideritis syriaca* subsp. *syriaca* has already become a crop locally in Crete with established value chains [35,37,38]. All these cases of unique Cretan NUPs bring to light the fact that new agro-alimentary products can be potentially sourced from these local endemic NUPs, if research gaps are bridged, marketing is successfully engaged, and stakeholder attraction is carefully attained [22]. Additionally, these unique NUPs can possibly exploit extant value chains of related, but commonplace, plant products, namely those of Greek oregano [*Origanum vulgare* L. subsp. *hirtum* (Link) A. Terracc.], Turkish oregano (*Origanum onites* L.), Spanish oregano [*Thymbra capitata* (L.) Cav.], marjoram (*Origanum majorana*), Greek mountain tea or shepherds’ tea (*Sideritis* spp.), everlasting or curry plant [*Helichrysum italicum* (Roth) G. Don], catmint (*Nepeta cataria* L.), etc.

The ability of modern technologies to transform harvested crops into a range of diverse products and uses with an extended shelf-life may create, nowadays, new opportunities to market novel products [7]. Local endemic NUPs such as those evaluated herein have a strong potential to shape a unique and solid product identity of local character (potential new products with protected designation of origin) which can also be exploited in terms of exclusive marketing, if well-protected legally [22]. In order to advertise new plant products based on, or sourced from, local endemic NUPs or popularize new uses for them, the development and promotion of user guides and recipe books, both in local and foreign languages, is required [8]. Top chefs, popular restaurants, TV shows, social media, and known food retailers can play a leading role in promoting and establishing new uses of NUPs, especially in nutrition, gastronomy, and food systems that occupy a great part of our daily livelihoods [8,28]. New culinary uses may also be documented and established even for plants never used before traditionally for strict culinary preparations, just as it was developed for the case *Origanum dictamnus* [28], as well as for *Origanum microphyllum* (a close relative of marjoram) and *Sideritis syriaca* subsp. *syriaca* [35]. For these Cretan local endemic NUPs, new culinary preparations have been introduced recently using their beneficial herbal teas, suggesting to incorporate them into standard Mediterranean meal preparations for an enhanced beneficial effect [28,35]. This trend actually represents a contemporary approach to the ancient and world famous Mediterranean nutrition, inspiring the enrichment of everyday food preparations with the beneficial health effects of EU approved traditional herbal medicines, such as *Origanum dictamnus* [36] and *Sideritis* spp. [37].

### 3.3. Readiness Timescale for Sustainable Exploitation of the Focal NUPs in the Agro-Alimentary Sector

Previous SWOT and gap analyses indicate that, in order to devise or to create new value chains in any economic sector for NUPs (such as the local endemic NUPs studied herein with respect to the agro-alimentary sector), five general conditions should first be accomplished as necessary prerequisites [22], i.e., extant high agro-alimentary potential; unique product identity; availability of propagation material with Access and Benefit-Sharing (ABS) mechanisms already in place (Nagoya Protocol, EU Directive 511/2014); propagation and cultivation techniques in place and adequate research already conducted; incorporated commercial interest (or triggered interest) able to attract stakeholders, and extant distribution channels. Among all the local endemics of all regions/countries (n = 399 taxa), the readiness timescale for sustainable exploitation was indeterminable in 67.67% of the cases (280 taxa) and determinable for only 119 taxa (29.82%) [22]. Among the top 15 of the local endemic NUPs with above average agro-alimentary potential in each of the three regions/countries, the readiness timescale for sustainable exploitation was determinable in only 33.33% of the cases, while for 66.67% of the taxa it was indeterminable. 

Among the top 15 taxa evaluated as promising in the agro-alimentary sector, the readiness timescale was assessed as already achieved only in the case of *Origanum dictamnus* (Lamiaceae), a local Cretan endemic. The readiness timescale was designated as achievable in the short-term for 5 of the Cretan taxa (*Calamintha cretica, Helichrysum heldreichii, Nepeta sphaciotica, Sideritis syriaca* subsp. *syriaca, Thymbra calostachya*) and in the medium-term for *Origanum microphyllum*. The readiness timescale for *Sideritis syriaca* subsp. *syriaca* should also be considered as ‘already achieved’ based on the recently filled research gaps [38]. The best example-cases of local endemic Cretan plants with optimum evaluated agro-alimentary potential are illustrated in Table 1.

The readiness timescale for the Moroccan (Mediterranean coast-Rif) *Centaurium erythraea* subsp. *bifrons* and *Abies marocana* as well as for the Tunisian *Artemisia campestris* subsp. *cinerea* was designated as achievable in the long-term (Supplementary Material S2). It seems that good chances are present for them if research gaps are filled promptly. *Argania spinosa* (L.) Skeels (Sapotaceae) represents a successful example of promotion of an endemic NUP of south-western Morocco and Algeria (however, not occurring in the Mediterranean coast and Rif studied herein). A. *spinosa* was traditionally used as food for centuries but was neglected and underutilized both locally and worldwide. After targeted research by scientists, and the documentation of its potential in the cosmetic sector, significant conservation and development efforts have been multiplied at local, regional and national scales, and these have opened the doors for the international markets [39]. At local scales, relevant studies [39] report the establishment of a local economic interest group for the development, preservation, and valorization of the argan forest of Morocco, promoting the optimization of women’s work, the protection and maintenance of existing *A. spinosa* trees, the plantation of young trees, and the promotion of new and innovative products. Last, but not least, and acknowledging the current international value of the previously considered NUP *A. spinosa,* the United Nations recently decided to declare May 10 as the International Argan Day, which will be celebrated annually.

## 4. Materials and Methods

### 4.1. Study Area and Target-Plants

The study area of this work covers the island of Crete (Greece), the Mediterranean coast-Rif of Morocco and Tunisia (whole national territory). The catalogue of the local endemic plants studied herein (unique floristic elements of these areas thriving nowhere else) includes 399 taxa (species and subspecies), i.e., 223 single-island local endemic plant taxa of Crete (Greece) [29], 94 single-region endemic taxa of Rif and the Mediterranean coast of Morocco, as well as 82 single-country endemic taxa of Tunisia [34]. 

### 4.2. Methodological Scheme Applied

In the frame of the MULTI-VAL-END project (ARIMNet2), a group of 13 research scientists with complementary expertise see [22] from Greece, Morocco and Tunisia have conducted several workshops and meetings to develop a new methodology for the evaluation of NUPs in the agro-alimentary sector. This scheme was applied to the focal taxa of the study area. After detailed discussion and examination of the potential advantages and disadvantages related the attributes and their possible scoring, the members of the consortium adopted 19 attributes in total to be used for the evaluation of the targeted single-region and single-country endemic taxa (n = 399) in the agro-alimentary sector (Table 2). Among the 19 selected attributes, seven were assessed as sector-specific (Table 2), reflecting explicit interest concerning the specific potential of the target taxa in the agro-alimentary sector (Level I evaluation), while 12 of the attributes were employed as prerequisites of common interest across various economic sectors (e.g., agro-alimentary, ornamental-horticultural, medicinal-cosmetics sectors), thus facilitating the sustainable exploitation of the target-taxa (Level II evaluation) [22].

Up to four types of data sources per attribute were prioritized for the evaluation, i.e., literature survey, best expert judgment, survey over internet sources and interviews with elderly people (Supplementary Material S1). In five cases of attributes (food additive potential, beverage potential, aromatic properties, wild edible greens, and bee attraction), all four types of sources were used for the evaluation of taxa; in two cases of attributes (type of aroma and spicy element), three of them were consulted to score each taxon. The most common data source used was internet survey and best expert judgment. During the scoring of each attribute in the agro-alimentary sector, the experts of each country have reviewed and prepared in advance a list of selected data sources per attribute, thus facilitating the later stages of evaluation.

After consultation with the members of the consortium, the scaling for each attribute was defined (three-fold to five-fold), and this was based on the quality and quantity of extant information for every taxon and the concomitant possible score value. The scoring of each attribute was based on the relevance of the information obtained from the analysis of existing data. Therefore, one attribute allowed a three-grade scale (3 possible scores); two attributes were on four-grade scale and four attributes allowed five (5) possible scores (Table 2).

Through co-creation procedures [22], the directionality of attribute scaling and scoring values were designated, indicating the interesting and/or desired characteristics and/or the strong values per attribute for each studied plant taxon. According to best expert judgment, lower attribute score was always assigned to cases of taxa with an absence of data, undesired characteristics, and/or absence of values, while higher scores were assigned to cases of taxa with desired characteristics, and/or very interesting features. To apply the above-mentioned methodological scheme, three end-users with academic education (Bachelor and Master of Science) and/or PhD were recruited from the local academic environments in each country. During tutorials, they consulted the relevant information per attribute prepared by the task force following the guidelines given, and they scored independently the target-taxa of the three regions. The scoring procedure was completed in repetitive detached sessions, considering only one or few related attributes or one taxon at a time. In this way, all attributes and/or taxa of all three focal regions were progressively scored. By scoring completion per region, the datasets created were checked for consistency, and they were revised by the project’s experts.

### 4.3. Evaluation Levels

**Level I evaluation (L-I)**: At the first level, the agro-alimentary potential of each local endemic taxon was evaluated using a point scoring system with seven sector-specific attributes (Table 2). Examples of scoring of taxa along with guidelines and sources consulted are given in Supplementary Material S1. The sum of scorings for all attributes was calculated and it was expressed as relative percentage (%) of the maximum possible score that could be generated in the agro-alimentary sector, i.e., sum of maximum scores for all attributes. To illustrate the most interesting/promising taxa per country for the agro-alimentary sector, three lists of hierarchically ranked taxa per country were produced (see Supplementary Material S2).

**Level II evaluation (L-II):** The feasibility for the sustainable exploitation of the endemic plants of Crete (Greece), Mediterranean coast-Rif of Morocco and Tunisia (Level II evaluation, L-II) is based on 12 attributes described in [22] as prerequisites of common interest across various economic sectors. Eight of these attributes represent the pre-conditions that should be met prior to any sustainable exploitation of the target taxa in any economic sector (also in the agro-alimentary sector), i.e., available initial plant material for propagation as well as species-specific propagation and cultivation techniques [22]. The remaining four attributes outline the special plant features and identity elements that could be exploited in product branding and marketing, thus facilitating trade exclusiveness, i.e., taxon’s endemism or uniqueness, rarity, extinction risk, and protection statuses [22]. The sum of scorings for all these attributes outlines the most feasible cases for sustainable exploitation of taxa in an economic sector [22], thus also applying in the agro-alimentary sector.

**Level III evaluation, L-III:** The readiness timescale for value chain creation regarding the focal taxa (Level III evaluation) is based on the SWOT (Strengths, Weaknesses, Opportunities, Threats) and gap analyses performed in own previous research [22]. In brief, eight parameters are involved in the evaluations, i.e., feasibility ranking class (L-II), potential for up-scaling to address commercial demand, availability of propagation material, possibility to overcome legal restrictions on ABS (in relation to Interest), overview of extant research (research gaps), estimated attraction of new producers, and retailers, estimated difficulty for value chain creation, and estimated exploitation of distribution channels [22]. These criteria are applied in each case (focal taxon), and a single characterization is designated [22]. This evaluation allows determining if the sustainable exploitation in the agro-alimentary (or other) sector has already been achieved in some cases of taxa, whether this is indeterminable, or if it is achievable in short-term, medium-term, and long-term [22]. 

### 4.4. Statistical Analysis

To explore how the different agro-alimentary attributes and focal taxa are grouped in each study region, we performed complete linkage hierarchical cluster analyses with 1- Pearson r distance measure based on the individual scores of each of the endemic taxa for the seven selected agro-alimentary attributes. This type of analysis is aimed to examine possible patterns in the studied regions regarding the different dimensions of the agro-alimentary interest of the focal NUP taxa and how these are discerned or grouped together.

## 5. Conclusions

This study introduces a new methodological scheme for the multifaceted agro-alimentary evaluation of NUPs, focusing on 399 unique floristic elements (single-region or single-country endemic plants) of three Mediterranean regions (Crete, Greece; Mediterranean coast-Rif of Morocco; Tunisia). Although more research work and stakeholder attention is certainly needed to unlock the full potential of the evaluated herein local endemic NUPs, this study produced hierarchical ranking of their agro-alimentary potential and discussed feasibility and readiness timescale assessments for their sustainable exploitation. 

In general, more effective uses of NUPs could support more nutrition-sensitive, resilient, and sustainable agro-alimentary systems. However, coordinated action, as well as basic and applied research, is needed to address many challenges such as domestication and ex-situ conservation concerns, breeding issues, poor consumer appeal, non-extant market niches or low market prices, unknown or difficult agro-processing, and compromised in-situ conservation of these NUPs [40,41], as these are often threatened by habitat degradation and human activities [12,32,33]. Yet, NUPs can help to increase the diversification of food production, adding new species to our diets with beneficial properties. To introduce local endemic NUPS in the agro-alimentary sector, the development of species-specific propagation and cultivation techniques is indispensable, improved cultivars should be aimed for in the future, and the development of new products that are able to attract stakeholders and extant distribution channels are required. 

## Figures and Tables

**Table 1:** Top cases of local endemic Cretan plants with strong agro-alimentary potential (Level I evaluation) associated with high feasibility and readiness timescale for sustainable exploitation (Level II and III evaluations, after [22]).

Taxon	Agro-Alimentary Potential (I)	Sustainable Exploitation Feasibility (II)	Readiness Timescale (III)
*Origanum dictamnus*	80.95%	91.67%	Already achieved
*Sideritis syriaca* subsp. *syriaca*	80.95%	66.67%	Short-term (or achieved)
*Origanum microphyllum*	80.95%	52.78%	Medium-term

**Table 2:** Sector-specific attributes and score values selected for the evaluation of the agro-alimentary potential (Level I evaluation, L-I) of the local endemic plants of Crete (Greece), Rif and Mediterranean coast of Morocco, and Tunisia, outlining the escalation of interest and the directionality of scoring. For examples on scoring of the studied taxa, guidelines and data sources used see Supplementary Material S1. For scoring of attributes of Levels II and III, see [22]).

Attribute	Short Description	Score 0	Score 1	Score 2	Score 3	Score 4	Score 5	Score 6	Possible Scores
**Food additive potential**	Use for alimentary or flavouring purposes	No	-	-	Under investigation	Possible	-	Yes	0, 3, 4, 6
**Beverage potential**	Use as tea infusion, tisane or decoction	No	-	-	Under investigation	Possible	-	Yes	0, 3, 4, 6
**Aromatic properties**	Perceived volatile constituents	No	Uncertain/Ambiguous	-	-	-	-	Yes	0, 1, 6
**Type of aroma**	Perceived basic aroma type	Bad or not smelling	Other	-	Indifferent/Light	Pungent	-	Pleasant	0, 1, 3, 4, 6
**Wild edible green**	Alimentary value as wild edible plant	No	Uncertain/Ambiguous	-	Under investigation	Possible	-	Yes	0, 1, 3, 4, 6
**Spicy element**	Savoury, flavouring and/or beneficial value	No	Uncertain/Ambiguous	-	Under investigation	Possible	-	Yes	0, 1, 3, 4, 6
**Bee attraction**	Apicultural nutritional value	No	Uncertain/Ambiguous	-	Under investigation	Possible	-	Yes	0, 1, 3, 4, 6

## Data Availability

The data presented in this study are available on request from the corresponding authors.

## Supplementary Materials

The following are available online at https://www.mdpi.com/article/10.3390/plants10091770/s1, S1: Level I evaluation examples in the agro-alimentary sector related to local endemic plants of Crete, Greece (223 taxa), Mediterranean coast-Rif of Morocco (94 taxa) and Tunisia (92 taxa) with explanations, guidelines, data sources used, and scoring per attribute. S2: Hierarchically arranged percentages (%) of the maximum possible scores achieved regarding the agro-alimentary potential of the local endemic plants of Crete, Rif-Mediterranean coast of Morocco and Tunisia indicating blue cell cases (>70%), green cell cases (>55–70%), yellow cell cases (>50–55%), grey cell cases (>35–50%) and no colour cases (<35%) in combination with Level II (Sustainable exploitation feasibility) and Level III (Readiness timescale for sustainable exploitation) multifaceted evaluations [22].
