# Supplementary material for: Agro-Alimentary Potential of the Neglected and Underutilized Local Endemic Plants of Crete (Greece), Rif-Mediterranean Coast of Morocco and Tunisia: Perspectives and Challenges
Source: Plants (Basel). 2021 Aug 25;10(9):1770. doi: 10.3390/plants10091770 (PMC8469186; doi:10.3390/plants10091770)
Supplement: Supplementary file 1 [file plants-10-01770-s001.zip › plants-1350393-supplementary/_Supplementary material S1 - agroalimentary.pdf]

Supplementary material S1: Level I evaluation examples in the agro-alimentary sector related to local endemic plants of Crete, Greece (223 taxa), Mediterranean coast-Rif of Morocco (94 taxa) and Tunisia (92 taxa) with explanations, guidelines, data sources used, and scoring per attribute

Level I – Evaluation of the potential of a taxon in the agro-alimentary sector

Attribute 1: Food additive potential (Use for alimentary or flavouring purposes)
Guidelines: Review botanical, pharmacognostic literature and enthnobotanical data; additionally, consultation with experts and/or interviews with elderly people / Yes = documented use as food, spice or condiment; Possible = other similar species in the same genus are known for edible uses; Under investigation = Test/Study in progress; No = Unknown or no data
Data sources: Juan et al. 2004, EMA 2013, 2015, 2016; PFAF 2021, best expert judgment and estimation based on literature.
Score 6: Taxa that are traditionally or currently used as food additives, e.g., *Origanum microphyllum, O. dictamnus* (EMA 2013, Krigas et al. 2015, Stikoudi et al. 2016) and *Centaurium erythraea* subsp. *bifrons* (EMA 2015). **Score 4:** Taxa that are potentially used as food additives, e.g., *Allium bourgeaui* subsp. *creticum*, *Muscari spreitzenhoferi* and *Teucrium gypsophilum*. **Score 3:** Taxa with suspected food additive value, e.g., *Helichrysum heldreichii*. **Score 0:** Taxa without known food additive value, e.g., *Bellevalia sitiaca* and *Festuca rifana*.

Attribute 2: Beverage potential (Use as tea infusion, tisane or decoction)
Guidelines: Review botanical, pharmacognostic literature and enthnobotanical data; additionally, consultation with experts and/or interviews with elderly people / Yes = documented use; Possible = known use in other similar species of the same genus (congeners); Under investigation = only few have tried or test in progress (not well established use); No = No data
Data sources: EMA 2012, 2013, 2016, Krigas et al. 2015, Leonidou 2016, Stikoudi et al. 2016; additionally, best expert judgment and PFAF 2021.
Score 6: Taxa that are traditionally used as beverages, e.g., *Sideritis syriaca* subsp. *syriaca* (EMA 2016)*, Origanum dictamnus* (EMA 2014, 2016, Krigas et al. 2015, Leonidou et 2016, Stikoudi et al. 2016) and *Centaurium erythraea* subsp. *bifrons* (EMA 2012). **Score 4:** Taxa that are potentially used as beverages, e.g., *Origanum microphyllum* and *Stachys fontqueri* (Leonidou 2016). **Score 3:** Taxa with suspected beverages value, e.g., *Helichrysum doerfleri* (N. Krigas, personal communication based on a PhD thesis). **Score 0:** Taxa without known beverage value, e.g., *Anthemis filicaulis* and *Sonchus fragilis*.

Attribute 3: Aromatic properties (Perceived volatile constituents)
Guidelines: Review botanical, pharmacognostic literature and enthnobotanical data; additionally, consultation with experts and/or interviews with elderly people / Yes = aromatic character; Uncertain / Ambiguous = possibly aromatic or controversial data; No = No aromatic character
Data sources: Ait Igri, 1990; El Oualidi et al., 2002 and internet survey; Best expert judgment based on empirical knowledge

Score 6: Taxa with aromatic properties, e.g., *Abies marocana* (Ait Igri, 1990), *Teucrium grosii* and *Origanum dictamnus*. **Score 1:** Taxa with uncertain aromatic properties, e.g., *Ferulago thyrsiflora* and *Centaurium barrelieroides*. **Score 0:** Taxa without aromatic properties, e.g., *Tulipa cretica* and *Acis tingitana*.

Attribute 4: Type of aroma (Perceived basic type of aroma)
Guidelines and data sources: Based on literature sources or according to personal smell assessment of the evaluators; additionally, best expert judgement; Ait Igri, 1990 and internet survey.
Score 6: Taxa with pleasant aroma, e.g., *Origanum dictamnus, Abies marocana* and *Salvia interrupta* subsp. *paui*. **Score 4:** Taxa with pungent aroma, e.g., *Calamintha cretica*. **Score 3:** Taxa with indifferent-light aroma, e.g., *Teucrium alpestre*. **Score 1:** Taxa with another aroma compared to previous scores, e.g., *Scutellaria hirta and Xiphion rutherfordii*. **Score 0:** Taxa with bad smelling or without smelling, e.g., *Carlina diae*.

Attribute 5: Wild edible green (Alimentary value as wild edible green)
Guidelines: Review botanical and enthnobotanical data; additionally, consultation with experts and/or interviews with elderly people / Yes = documented use as wild edible green; Possible = known edible use in other similar species of the same genus (congeners); Under investigation = only few have tried or test in progress (not well established use); No = No data
Data sources: Crete - Athanasiou 2014, Koutsos 2015, Lampraki 2000, Papiomitoglou & Nikitidis 2011, Papoulias 2006, Psaroudaki 2012, PFAF 2021; additionally, best expert judgment based on similar edible species.
Score 6: Taxa with documented edible value, e.g., *Campanula pelviformis*. **Score 4:** Taxa that are potentially edible, e.g., *Allium platakisii, Malva vidalii* and *Rumex brachypodus*. **Score 3:** Taxa with suspected edible value (no case). **Score 1:** Taxa with uncertain-ambiguous edible value, e.g., *Ferulago thyrsiflora*. **Score 0:** Taxa without known edible value, e.g., *Carlina diae* and *Xiphion rutherfordii*.

Attribute 6: Spicy element (Savoury, flavouring and/or beneficial value)
Guidelines and data sources: Review botanical and enthnobotanical data; additionally, consultation with experts and/or interviews with elderly people / Yes = documented use as spicy element; Possible = known use as spice in other similar species of the same genus (congeners); Under investigation = only few have tried or test in progress (not well-established use); No = No data
Score 6: Taxa that are traditionally used as spice, e.g., *Origanum microphyllum* and *Centaurium erythraea* subsp. *bifrons*. **Score 4:** Taxa that can be potentially used as spice, e.g., *Thymbra calostachya* and *Teucrium rifanum*. **Score 3:** Taxa with suspected value as spicy elements (no case). **Score 1:** Taxa with uncertain-ambiguous value as spicy elements, e.g., *Nepeta sphaciotica*. **Score 0:** Taxa without documentation, e.g., *Anthemis samariensis* and *Vicia cedretorum*.

Attribute 7: Bee attraction (Apicultural value)
Guidelines: Review literature; additionally, consultation with experts and/or interviews with elderly people / Yes = Plant used for bee/pollinator attraction;

Possible = Similar species of the same genus (congener) is used for bee/pollinator attraction; Under investigation = Suspected bee/pollinator attraction or observation in progress; Uncertain/Ambiguous = Controversial information; No = No data
Data sources: Royal Horticultural Society (www.rhs.org.uk/science/conservation-biodiversity/wildlife/plants-for-pollinators)
Score 6: Taxa that could attract pollinators including bees, e.g., *Centaurea argentea* subsp. *chionantha, Centaurium erythraea* subsp. *bifrons* and *Teucrium gypsophilum*. **Score 4:** Taxa that could potentially attract pollinators including bees, e.g., *Convolvulus vidalii*. **Score 3:** Taxa for which pollinator/bee attraction ability is suspected (no case for Crete). **Score 1:** Taxa for which pollinator/bee attraction ability is uncertain, e.g., *Scorzonera mollis* subsp. *idaea*. **Score 0:** Taxa with no documentation on pollinator/bee attraction, e.g., *Cyclamen confusum* and *Festuca rifana*.

References

Ait Igri, M. Chemical composition of twigs and wood essential oils of *Abies pinsapo* Boiss. ssp. *marocana* (Trab.) Emb. and Maire. *AGRIS* **1990**, *5*, 7-11.

Athanasiou Z. Wild herbs and edible greens. Psychalos editions; Athens, 2014.

El Oualidi J.; Puech S.; Navarro T. Geographical variation and successive adaptative radiation of yellow-flowered *Teucrium* (Labiatae) in Mediterranean region. *Bot. Review* **2002**, *68*, 209-234.

EMA/HMPC. Final Assessment Report on *Origanum dictamnus* L., Herba (200431/2012); European Medicines Agency/Committee on Herbal Medicinal Products: London, UK, 2013; Available online: http://www.ema.europa.eu/docs/en_GB/document_library/ Herbal_-_HMPC_assessment_report/2013/08/WC500147179.pdf (accessed on 18 February 2021).

EMA/HMPC. Final Assessment Report on *Centaurium erythraea Rafn*. s.l., Herba (277491/2015); European Medicines Agency/Committee on Herbal Medicinal Products: London, UK, 2015; Available online: https://www.ema.europa.eu/en/documents/herbal-report/assessment-report-centaurium-erythraea-rafn-sl-herba_en.pdf (accessed on 18 February 2021).

EMA/HMPC. Final Assessment Report on *Sideritis scardica* Griseb.; *Sideritis clandestina* (Bory & Chaub.) Hayek; *Sideritis raeseri* Boiss. & Heldr.; *Sideritis syriaca* L., Herba (39455/2015); European Medicines Agency/Committee on Herbal Medicinal Products: London, UK, 2016; Available online: https://www.ema.europa.eu/en/documents/herbal-report/final-assessment-report-sideritis-scardica-griseb-sideritis-clandestina-bory-chaub-hayek-sideritis_en.pdf (accessed on 18 February 2021).

Juan, R.; Pastor, J.; Millán, F.; Alaiz, M., Vioque, J. Amino Acids Composition of Teucrium Nutlet Proteins and their Systematic Significance. *Ann. Bot.* **2004**, *94*, 615–621.

Koutsos, B.T. Vegetables from God's garden. Kyriakidis Editions, Thessaloniki, 2015.

Krigas, N.; Lazari, D.; Maloupa, E.; Stikoudi, M. Introducing Dittany of Crete (*Origanum dictamnus* L.) to gastronomy: A new culinary concept for a traditionally used medicinal plant. *Int. J. Gastron. Food Sci.* **2015**, *2*, 112-118.
Lampraki, M. The wild greens. Greek Letters Editions, Athens, 2000.
Leonidiou, F. Medicinal and aromatic plants of Greece: Inventory and conservation priorities. Master Thesis, Faculty of Crop Science, Agricultural University of Athens, 2016 [In Greek with English abstract].
Nikitidis, N., Papiomytoglou, V. Green plants & herbs of Greece. Mediterraneo Editions, Crete, Greece, 2011.
Papoulias, T. The wild edible greens of mountains and fields. Pyschalos Editions, Athens, 2006.
PFAF (Plants For A Future) 2021. https://pfaf.org (access on 18 February 2021)
Psaroudaki, A. 2012. Recording, botanical identification, genetic diversity, attributes of indigenous edible plants of Crete and their participation in the current nutritional pattern. Ph. D. Thesis, Faculty of Crop Science, Agricultural University of Athens, 2016 [In Greek with English abstract].
Stikoudi, M.; Maloupa, E.; Lazari, D.; Krigas, N. Aromagarden and Cooking for Wellness: Discovering secrets of Mediterranean plants. i-Print, Thessaloniki 2016. ISBN 978-960-16-6011-0

RBG, 2016. The State of the World's Plants Report 2016. Royal Botanic Gardens, Kew, UK. https://stateoftheworldsplants.org/2016/report/sotwp_2016.pdf (access on 6 February 2021).
