# Supplementary material for: Agro-Alimentary Potential of the Neglected and Underutilized Local Endemic Plants of Crete (Greece), Rif-Mediterranean Coast of Morocco and Tunisia: Perspectives and Challenges
Source: Plants (Basel). 2021 Aug 25;10(9):1770. doi: 10.3390/plants10091770 (PMC8469186; doi:10.3390/plants10091770)
Supplement: Supplementary file 1 [file plants-10-01770-s001.zip › plants-1350393-supplementary/_Supplementary material S2 - agroalimentary.pdf]

Supplementary material S2: Hierarchically arranged percentages (%) of the maximum possible scores achieved regarding the agro-alimentary potential of the local endemic plants of Crete, Rif-Mediterranean coast of Morocco and Tunisia indicating blue cell cases (>70%), green cell cases (>55-70%), yellow cell cases (>50-55%), grey cell cases (>35-50%) and no colour cases (<35%) in combination with Level II (Sustainable exploitation feasibility) and Level III (Readiness timescale for sustainable exploitation) multifaceted evaluations (Krigas et al. 2021).

A. Local endemic plants of Crete (Greece)

Taxa	Level I	Level II	Level III
Origanum dictamnus	85.71	91.67	Already achieved
Origanum microphyllum	80.95	52.78	Medium term
Sideritis syriaca subsp. *syriaca*	80.95	66.67	Short term
Thymbra calostachya	71.43	55.56	Short term
Helichrysum doerfleri	59.52	45.83	Long term
Helichrysum heldreichii	59.52	61.11	Long term
Calamintha cretica	59.52	69.44	Short term
Allium bourgeaui subsp. *creticum*	54.76	37.50	Long term
Allium circinnatum subsp. *circinnatum*	54.76	29.17	Indeterminable
Allium dilatatum	54.76	27.78	Indeterminable
Allium platakisii	54.76	41.67	Long term
Micromeria hispida	54.76	45.83	Long term
Micromeria sphaciotica	54.76	40.28	Long term
Nepeta sphaciotica	50.00	62.50	Short term
Prunella cretensis	50.00	34.72	Indeterminable
Teucrium cuneifolium	50.00	63.89	Short term
Crocus oreocreticus	45.24	41.67	Long term
Sanguisorba cretica	45.24	38.89	Long term
Centaurea idaea	42.86	36.11	Long term
Campanula pelviformis	42.86	43.06	Long term
Petromarula pinnata	42.86	45.83	Long term
Teucrium alpestre	42.86	36.11	Long term
Viola fragrans	40.48	34.72	Indeterminable
Onopordum bracteatum subsp. *creticum*	38.10	38.89	Long term
Dianthus fruticosus subsp. *creticus*	35.71	34.72	Indeterminable
Dianthus fruticosus subsp. *sitiacus*	35.71	30.56	Indeterminable
Dianthus juniperinus subsp. *aciphyllus*	35.71	33.33	Indeterminable
Dianthus juniperinus subsp. *bauhinorum*	35.71	51.39	Medium term
Dianthus juniperinus subsp. *heldreichii*	35.71	33.33	Indeterminable
Dianthus juniperinus subsp. *idaeus*	35.71	33.33	Indeterminable

Dianthus juniperinus subsp. *juniperinus*	35.71	33.33	Indeterminable
Dianthus juniperinus subsp. *kavusicus*	35.71	50.00	Medium term
Dianthus juniperinus subsp. *pulviniformis*	35.71	33.33	Indeterminable
Dianthus sphacioticus	35.71	34.72	Indeterminable
Dianthus xylorrhizus	35.71	58.33	Short term
Phlomis lanata	35.71	51.39	Medium term
Muscari spreitzenhoferi	33.33	37.50	Long term
Crepis auriculifolia	33.33	58.33	Short term
Crepis sibthorpiana	33.33	52.78	Medium term
Hypochaeris tenuiflora	33.33	33.33	Indeterminable
Lactuca alpestris	33.33	29.17	Indeterminable
Tragopogon lassithicus	33.33	50.00	Medium term
Campanula cretica	33.33	36.11	Long term
Limonium chersonesum	33.33	27.78	Indeterminable
Chaerophyllum creticum	30.95	52.78	Medium term
Scutellaria hirta	30.95	29.17	Indeterminable
Scutellaria sieberi	30.95	36.11	Long term
Bupleurum gaudianum	23.81	51.39	Medium term
Bupleurum kakiskalae	23.81	58.33	Short term
Eryngium ternatum	23.81	54.17	Medium term
Cirsium morinifolium	23.81	23.61	Indeterminable
Hieracium schmidtii subsp. *creticum*	23.81	22.22	Indeterminable
Klasea cretica	23.81	26.39	Indeterminable
Arabis cretica	23.81	29.17	Indeterminable
Noccaea cretica	23.81	34.72	Indeterminable
Noccaea zaffranii	23.81	41.67	Long term
Campanula creutzburgii	23.81	33.33	Indeterminable
Campanula hierapetrae	23.81	58.33	Short term
Campanula jacquinii	23.81	38.89	Long term
Campanula saxatilis subsp. *saxatilis*	23.81	59.72	Short term
Campanula spatulata subsp. *filicaulis*	23.81	23.61	Indeterminable
Campanula tubulosa	23.81	33.33	Indeterminable
Sedum eriocarpum subsp. *spathulifolium*	23.81	22.22	Indeterminable
Lathyrus neurolobus	23.81	41.67	Long term
Medicago arborea subsp. *strasseri*	23.81	50.00	Medium term
Ononis verae	23.81	27.78	Indeterminable
Trifolium phitosianum	23.81	18.06	Indeterminable
Crocus sieberi	23.81	33.33	Indeterminable
Galium extensum	23.81	27.78	Indeterminable
Galium fruticosum	23.81	44.44	Long term
Galium incanum subsp. *creticum*	23.81	44.44	Long term

Galium incrassatum	23.81	38.89	Long term
Galium incurvum	23.81	23.61	Indeterminable
Centranthus sieberi	23.81	44.44	Long term
Scorzonera mollis subsp. *idaea*	21.43	45.83	Long term
Viola alba subsp. *cretica*	19.05	23.61	Indeterminable
Geocaryum creticum	14.29	38.89	Long term
Horstrissea dolinicola	14.29	45.83	Long term
Arum idaeum	14.29	29.17	Indeterminable
Biarum davisii	14.29	41.67	Long term
Biarum tenuifolium subsp. *idomenaeum*	14.29	18.06	Indeterminable
Vincetoxicum creticum	14.29	38.89	Long term
Bellevalia brevipedicellata	14.29	62.50	Short term
Bellevalia juliana	14.29	44.44	Long term
Bellevalia sitiaca	14.29	38.89	Long term
Ornithogalum dictaeum subsp. *dictaeum*	14.29	33.33	Indeterminable
Ornithogalum insulare	14.29	22.22	Indeterminable
Prospero battagliae	14.29	15.28	Indeterminable
Prospero depressum	14.29	31.94	Indeterminable
Prospero hierapytnense	14.29	22.22	Indeterminable
Prospero idaeum	14.29	31.94	Indeterminable
Prospero rhadamanthi	14.29	38.89	Long term
Prospero talosii	14.29	33.33	Indeterminable
Scilla nana subsp. *albescens*	14.29	23.61	Indeterminable
Scilla nana subsp. *nana*	14.29	20.83	Indeterminable
Anthemis abrotanifolia	14.29	38.89	Long term
Anthemis filicaulis	14.29	33.33	Indeterminable
Anthemis glaberrima	14.29	62.50	Short term
Anthemis samariensis	14.29	33.33	Indeterminable
Anthemis tomentella	14.29	33.33	Indeterminable
Carlina diae	14.29	58.33	Short term
Centaurea argentea subsp. *chionantha*	14.29	45.83	Long term
Centaurea argentea subsp. *macrothysana*	14.29	33.33	Indeterminable
Centaurea baldaccii	14.29	36.11	Long term
Centaurea lancifolia	14.29	52.78	Medium term
Centaurea poculatoris	14.29	61.11	Short term
Centaurea redempta subsp. *redempta*	14.29	33.33	Indeterminable
Filago wagenitziana	14.29	29.17	Indeterminable
Inula candida subsp. *decalvans*	14.29	44.44	Long term
Phagnalon pygmaeum	14.29	27.78	Indeterminable
Senecio fruticulosus	14.29	34.72	Indeterminable
Staehelina petiolata	14.29	54.17	Medium term

Alkanna sieberi	14.29	37.50	Long term
Anchusa cespitosa	14.29	50.00	Medium term
Cynoglossum sphacioticum	14.29	40.28	Long term
Myosotis solange	14.29	40.28	Long term
Alyssum fragillimum	14.29	44.44	Long term
Alyssum idaeum	14.29	51.39	Medium term
Alyssum lassiticum	14.29	38.89	Long term
Alyssum sphacioticum	14.29	50.00	Medium term
Draba cretica	14.29	36.11	Long term
Erysimum creticum	14.29	44.44	Long term
Erysimum mutabile	14.29	29.17	Indeterminable
Erysimum raulinii	14.29	40.28	Long term
Ricotia cretica	14.29	34.72	Indeterminable
Bolanthus creutzburgii subsp. *creutzburgii*	14.29	27.78	Indeterminable
Bolanthus creutzburgii subsp. *zaffranii*	14.29	44.44	Long term
Bufonia stricta subsp. *cecconiana*	14.29	23.61	Indeterminable
Cerastium brachypetalum subsp. *doerfleri*	14.29	45.83	Long term
Cerastium deschatresii	14.29	23.61	Indeterminable
Cerastium scaposum subsp. *peninsularum*	14.29	26.39	Indeterminable
Cerastium scaposum subsp. *scaposum*	14.29	26.39	Indeterminable
Cherleria wettsteinii	14.29	34.72	Indeterminable
Petrorhagia candica	14.29	37.50	Long term
Petrorhagia dianthoides	14.29	47.22	Long term
Silene ammophila subsp. *ammophila*	14.29	52.78	Medium term
Silene antri-jovis	14.29	40.28	Long term
Silene flavescens subsp. *dictaea*	14.29	51.39	Medium term
Silene integripetala subsp. *greuteri*	14.29	37.50	Long term
Silene pinetorum subsp. *pinetorum*	14.29	40.28	Long term
Silene pinetorum subsp. *sphaciotica*	14.29	40.28	Long term
Silene sieberi	14.29	36.11	Long term
Silene variegata	14.29	29.17	Indeterminable
Telephium imperati subsp. *pauciflorum*	14.29	18.06	Indeterminable
Colchicum cretense	14.29	29.17	Indeterminable
Convolvulus argyrothamnos	14.29	59.72	Short term
Cuscuta atrans	14.29	43.06	Long term
Euphorbia rechingeri	14.29	44.44	Long term
Euphorbia sultan-hassei	14.29	37.50	Long term
Astragalus creticus subsp. *creticus*	14.29	22.22	Indeterminable
Astragalus creticus subsp. *minoicus*	14.29	23.61	Indeterminable
Astragalus dolinicola	14.29	29.17	Indeterminable
Astragalus idaeus	14.29	50.00	Medium term

Astragalus nummularius subsp. *nummularius*	14.29	27.78	Indeterminable
Ebenus cretica	14.29	45.83	Long term
Onobrychis sphaciotica	14.29	52.78	Medium term
Securigera globosa	14.29	43.06	Long term
Hypericum aciferum	14.29	48.61	Long term
Hypericum amblycalyx	14.29	33.33	Indeterminable
Hypericum empetrifolium subsp. *tortuosum*	14.29	27.78	Indeterminable
Hypericum jovis	14.29	56.94	Short term
Hypericum kelleri	14.29	48.61	Long term
Hypericum trichocaulon	14.29	29.17	Indeterminable
Acantholimon androsaceum	14.29	33.33	Indeterminable
Limonium calliopsium	14.29	47.22	Long term
Limonium chrisianum	14.29	27.78	Indeterminable
Limonium cornarianum	14.29	47.22	Long term
Limonium creticum	14.29	52.78	Medium term
Limonium elaphonisicum	14.29	50.00	Medium term
Limonium grabusae	14.29	27.78	Indeterminable
Limonium hierapetrae	14.29	33.33	Indeterminable
Limonium minoicum	14.29	27.78	Indeterminable
Limonium recticaule	14.29	27.78	Indeterminable
Limonium sougiae	14.29	29.17	Indeterminable
Limonium xerocamposicum	14.29	27.78	Indeterminable
Adonis cretica	14.29	22.22	Indeterminable
Clematis elisabethae-carolae	14.29	44.44	Long term
Ranunculus cupreus	14.29	38.89	Long term
Ranunculus radinotrichus	14.29	34.72	Indeterminable
Ranunculus veronicae	14.29	41.67	Long term
Cotoneaster creticus	14.29	33.33	Indeterminable
Asperula crassula	14.29	48.61	Long term
Asperula idaea	14.29	38.89	Long term
Asperula pubescens	14.29	33.33	Indeterminable
Asperula rigida	14.29	33.33	Indeterminable
Verbascum arcturus	14.29	47.22	Long term
Verbascum spinosum	14.29	40.28	Long term
Tamarix minoa	14.29	30.56	Indeterminable
Ferulago thyrsiflora	9.52	37.50	Long term
Gagea omalensis	9.52	27.78	Indeterminable
Anacamptis papilionacea subsp. *alibertis*	9.52	22.22	Indeterminable
Orchis sitiaca	9.52	25.00	Indeterminable
Orchis spitzelii subsp. *nitidifolia*	9.52	25.00	Indeterminable
Asplenium creticum	0.00	27.78	Indeterminable

Carex cretica	0.00	25.00	Indeterminable
Carex idaea	0.00	19.44	Indeterminable
Lomelosia albocincta	0.00	38.89	Long term
Lomelosia minoana subsp. *asterusica*	0.00	50.00	Medium term
Lomelosia minoana subsp. *minoana*	0.00	40.28	Long term
Lomelosia sphaciotica subsp. *decalvans*	0.00	27.78	Indeterminable
Lomelosia sphaciotica subsp. *sphaciotica*	0.00	27.78	Indeterminable
Corydalis uniflora	0.00	20.83	Indeterminable
Fritillaria messanensis subsp. *sphaciotica*	0.00	18.06	Indeterminable
Tulipa bakeri	0.00	41.67	Long term
Tulipa cretica	0.00	41.67	Long term
Tulipa doerfleri	0.00	40.28	Long term
Linum caespitosum	0.00	27.78	Indeterminable
Linum doerfleri	0.00	38.89	Long term
Cephalanthera cucullata	0.00	43.06	Long term
Epipactis cretica	0.00	25.00	Indeterminable
Himantoglossum samariense	0.00	18.06	Indeterminable
Serapias cordigera subsp. *cretica*	0.00	19.44	Indeterminable
Brachypodium sylvaticum subsp. *creticum*	0.00	34.72	Indeterminable
Catapodium borgesii	0.00	27.78	Indeterminable
Dactylis glomerata subsp. *rigida*	0.00	37.50	Long term
Festuca polita subsp. *cretica*	0.00	18.06	Indeterminable
Sesleria doerfleri	0.00	56.94	Short term
Polygonum idaeum	0.00	47.22	Long term
Cyclamen confusum	0.00	31.94	Indeterminable
Cyclamen graecum subsp. *candicum*	0.00	44.44	Long term
Viscum album subsp. *creticum*	0.00	16.67	Indeterminable
Zelkova abelicea	0.00	52.78	Medium term
Chaenorhinum idaeum	0.00	34.72	Indeterminable

B. Local endemic plants of Morocco (Rif-Mediterranean coast)

Taxa	Level I	Level II	Level III
Centaurium erythraea subsp. *bifrons*	71.43	40.28	Long term
Teucrium afrum subsp. *rubriflorum*	71.43	23.61	Indeterminable
Teucrium grosii	71.43	26.39	Indeterminable
Teucrium gypsophilum	71.43	30.56	Indeterminable

Teucrium huotii	71.43	25.00	Indeterminable
Teucrium rotundifolium subsp. *sanguisorbifolium*	71.43	19.44	Indeterminable
Teucrium chlorostachyum subsp. *chlorostachyum*	66.67	30.56	Indeterminable
Teucrium chlorostachyum subsp. *melillense*	66.67	30.56	Indeterminable
Teucrium rifanum	66.67	29.17	Indeterminable
Salvia interrupta subsp. *paui*	61.90	25.00	Indeterminable
Abies marocana	47.62	43.06	Long term
Centaurium barrelieroides	45.24	22.22	Indeterminable
Marrubium fontianum	42.86	22.22	Indeterminable
Marrubium heterocladum	42.86	23.61	Indeterminable
Anthemis mauritiana subsp. *mauritiana*	38.10	29.17	Indeterminable
Origanum elongatum	38.10	23.61	Indeterminable
Stachys fontqueri	38.10	18.06	Indeterminable
Vicia cedretorum	38.10	22.22	Indeterminable
Cytisus grandiflorus subsp. *haplophyllus*	33.33	12.50	Indeterminable
Cytisus striatus subsp. *megalanthus*	33.33	9.72	Indeterminable
Genista carpetana subsp. *nociva*	33.33	23.61	Indeterminable
Genista osmariensis	33.33	25.00	Indeterminable
Genista oxycedrina	33.33	19.44	Indeterminable
Malva vidalii	33.33	30.56	Indeterminable
Sonchus masguindalii	33.33	36.11	Long term
Centaurea alibeyana	28.57	25.00	Indeterminable
Centaurea monticola subsp. *orthoacantha*	28.57	22.22	Indeterminable
Centaurea oriolii-bolosii	28.57	22.22	Indeterminable
Centaurea xaveri	28.57	25.00	Indeterminable
Ononis alopecuroides subsp. *simulata*	28.57	30.56	Indeterminable
Ononis gines-lopezii	28.57	13.89	Indeterminable
Rumex brachypodus	28.57	18.06	Indeterminable
Silene abietum	28.57	22.22	Indeterminable
Silene lagrangei	28.57	25.00	Indeterminable
Silene rhiphaena	28.57	30.56	Indeterminable
Silene vidaliana	28.57	22.22	Indeterminable
Sonchus briquetianus	28.57	22.22	Indeterminable
Sonchus fragilis	28.57	25.00	Indeterminable
Trifolium tastetii	28.57	15.28	Indeterminable
Scrophularia fontqueri	26.19	25.00	Indeterminable
Scrophularia eriocalyx	21.43	22.22	Indeterminable
Antirrhinum martenii	19.05	30.56	Indeterminable
Arabis pubescens subsp. *leucanthemifolia*	19.05	13.89	Indeterminable
Armeria alpinifolia	19.05	30.56	Indeterminable

Convolvulus vidalii	19.05	29.17	Indeterminable
Eryngium caespitiferum	19.05	25.00	Indeterminable
Ferula bolivari	19.05	22.22	Indeterminable
Ferula fontqueri	19.05	13.89	Indeterminable
Fumaria macrosepala subsp. *megasepala*	19.05	27.78	Indeterminable
Fumaria macrosepala subsp. *obscura*	19.05	27.78	Indeterminable
Fumaria ouezzanensis subsp. *ramosa*	19.05	26.39	Indeterminable
Fumaria sepium subsp. *rugosa*	19.05	26.39	Indeterminable
Hypericum andjerinum	19.05	25.00	Indeterminable
Iberis contracta subsp. *rifana*	19.05	18.06	Indeterminable
Iberis grosmiquelii	19.05	22.22	Indeterminable
Lathyrus coerulescens	19.05	13.89	Indeterminable
Linum austriacum subsp. *gomaricum*	19.05	22.22	Indeterminable
Matthiola masguindalii	19.05	18.06	Indeterminable
Paronychia maroccana	19.05	23.61	Indeterminable
Saxifraga maweana	19.05	23.61	Indeterminable
Saxifraga rigoi subsp. *maroccana*	19.05	30.56	Indeterminable
Saxifraga tricrenata	19.05	31.94	Indeterminable
Saxifraga werneri	19.05	33.33	Indeterminable
Sedum mucizonia subsp. *abylaea*	19.05	18.06	Indeterminable
Sideritis arborescens subsp. *maireana*	19.05	16.67	Indeterminable
Sideritis romoi	19.05	16.67	Indeterminable
Xiphion rutherfordii	19.05	36.11	Long term
Helianthemum grosii	14.29	30.56	Indeterminable
Leucanthemopsis longipectinata	14.29	13.89	Indeterminable
Narcissus albimarginatus	14.29	16.67	Indeterminable
Rhodanthemum hosmariense	14.29	36.11	Long term
Acis tingitana	9.52	31.94	Indeterminable
Ammiopsis daucoides	9.52	8.33	Indeterminable
Anthyllis vulneraria subsp. *fatmae*	9.52	38.89	Long term
Anthyllis vulneraria subsp. *ajmasiana*	9.52	30.56	Indeterminable
Astragalus meuselii	9.52	18.06	Indeterminable
Astragalus tetuanensis	9.52	26.39	Indeterminable
Carduus martinezii subsp. *fontqueri*	9.52	22.22	Indeterminable
Cephalaria mauritanica subsp. *rifana*	9.52	20.83	Indeterminable
Echium canum	9.52	22.22	Indeterminable
Fumana fontqueri	9.52	16.67	Indeterminable
Hieracium riofrioi	9.52	22.22	Indeterminable
Mantisalca amberboides	9.52	8.33	Indeterminable
Ptilostemon abylensis	9.52	20.83	Indeterminable
Santolina adscensionis	9.52	16.67	Indeterminable

Bromopsis maroccana	0.00	30.56	
Bromus erectus subsp. *microchaetus*	0.00	38.89	Long term
Festuca embergeri	0.00	8.33	Indeterminable
Festuca fontqueriana	0.00	12.50	Indeterminable
Festuca rifana	0.00	18.06	Indeterminable
Hemicrambe fruticulosa	0.00	29.17	Indeterminable
Herniaria boissieri subsp. *maroccana*	0.00	29.17	Indeterminable
Moehringia glochidisperma	0.00	25.00	Indeterminable
Rhodalsine senneniana	0.00	25.00	Indeterminable

C. Local endemic plants of Tunisia

Taxa	**Level I**	**Level II**	**Level III**
Marrubium aschersonii	76.19	26.39	Indeterminable
Teucrium alopecurus	71.43	27.78	Indeterminable
Teucrium luteum subsp. *gabesianum*	71.43	25.00	Indeterminable
Artemisia campestris subsp. *cinerea*	66.67	50.00	Medium term
Teucrium nablii	47.62	30.56	Indeterminable
Teucrium radicans	47.62	27.78	Indeterminable
Teucrium sauvagei	47.62	22.22	Indeterminable
Calendula suffruticosa subsp. *suffruticosa*	42.86	36.11	Long term
Dianthus cintranus subsp. *byzacenus*	42.86	19.44	Indeterminable
Dianthus rupicola subsp. *hermaeensis*	42.86	26.39	Indeterminable
Galium afropusillum	40.48	27.78	Indeterminable
Galium olivetorum	40.48	23.61	Indeterminable
Ferula tunetana	33.33	22.22	Indeterminable
Limonium boitardii	33.33	23.61	Indeterminable
Limonium byzacium	33.33	23.61	Indeterminable
Limonium cercinense	33.33	27.78	Indeterminable
Limonium clupeanum	33.33	27.78	Indeterminable
Limonium comosum	33.33	23.61	Indeterminable
Limonium confertum	33.33	25.00	Indeterminable
Limonium elfahsianum	33.33	27.78	Indeterminable
Limonium formosum	33.33	23.61	Indeterminable
Limonium hipponense	33.33	23.61	Indeterminable
Limonium intricatum	33.33	27.78	Indeterminable
Limonium kairouanum	33.33	27.78	Indeterminable
Limonium korbousense	33.33	27.78	Indeterminable
Limonium lacertosum	33.33	23.61	Indeterminable
Limonium menigense	33.33	27.78	Indeterminable

Limonium neapolense	33.33	27.78	Indeterminable
Limonium oblanceolatum	33.33	23.61	Indeterminable
Limonium punicum	33.33	23.61	Indeterminable
Limonium pyramidatum	33.33	27.78	Indeterminable
Limonium rubescens	33.33	23.61	Indeterminable
Limonium serratum	33.33	27.78	Indeterminable
Limonium tacapense	33.33	23.61	Indeterminable
Limonium thaenicum	33.33	27.78	Indeterminable
Limonium tritonianum	33.33	23.61	Indeterminable
Limonium xerophylum	33.33	27.78	Indeterminable
Limonium zembrae	33.33	27.78	Indeterminable
Limonium zeugitanum	33.33	27.78	Indeterminable
Centaurea sicula subsp. *kroumirensis*	30.95	20.83	Indeterminable
Clematis tunisiatica	30.95	20.83	Indeterminable
Crepis juvenalis	30.95	5.56	Indeterminable
Lathyrus brachyodon	30.95	20.83	Indeterminable
Linaria aegyptiaca subsp. *glutinosa*	30.95	27.78	Indeterminable
Linaria cossonii	30.95	20.83	Indeterminable
Linaria multicaulis subsp. *multicaulis*	30.95	13.89	Indeterminable
Linaria paradoxa	30.95	25.00	Indeterminable
Linaria pseudoviscosa	30.95	27.78	Indeterminable
Linaria reflexa subsp. *doumetii*	30.95	27.78	Indeterminable
Linaria virgata subsp. *syrtica*	30.95	22.22	Indeterminable
Linaria virgata subsp. *tunetana*	30.95	25.00	Indeterminable
Rumex tunetanus	30.95	31.94	Indeterminable
Silene barrattei	30.95	20.83	Indeterminable
Trifolium tunetanum	30.95	20.83	Indeterminable
Sideritis tunetana	26.19	22.22	Indeterminable
Helianthemum crassifolium subsp. *djeniense*	21.43	23.61	Indeterminable
Helianthemum virgatum subsp. *africanum*	21.43	20.83	Indeterminable
Lomelosia robertii	21.43	27.78	Indeterminable
Sixalix lehoueroui	21.43	27.78	Indeterminable
Sixalix thysdrusiana	21.43	18.06	Indeterminable
Thymelaea sempervirens	21.43	22.22	Indeterminable
Elaeoselinum tunetanum	14.29	16.67	Indeterminable
Bituminaria tunetana	14.29	27.78	Indeterminable
Arabis tunetana	11.90	12.50	Indeterminable
Astragalus asterias subsp. *aristidis*	11.90	19.44	Indeterminable
Bellevalia dolichophylla	11.90	27.78	Indeterminable
Bellevalia galitensis	11.90	27.78	Indeterminable
Delphinium sylvaticum subsp. *ichkeulianum*	11.90	27.78	Indeterminable

Hyacinthoides kroumiriensis	11.90	15.28	Indeterminable
Sporobolus tourneuxii	11.90	27.78	Indeterminable
Erysimum teppneri	4.76	22.22	Indeterminable
Anarrhinum brevifolium	2.38	11.11	Indeterminable
Erinacea schoenenbergeri	2.38	11.11	Indeterminable
Festuca scholziana	2.38	22.22	Indeterminable
Odontites citrinus	2.38	9.72	Indeterminable
Oncostema maireana	2.38	18.06	Indeterminable
Ophrys omegaifera subsp. *hayekii*	2.38	25.00	Indeterminable
Ophrys pseudomigoutiana	2.38	13.89	Indeterminable
Ophrys x carpitana	2.38	27.78	Indeterminable
Poa lehoueroui	2.38	16.67	Indeterminable
Serapias × debelairii	2.38	23.61	Indeterminable
Stipa alba	2.38	27.78	Indeterminable
